# Male partners’ support and influence on pregnant women’s oral PrEP use and adherence in Malawi

**DOI:** 10.3389/frph.2023.1206075

**Published:** 2023-08-08

**Authors:** Alinda M. Young, Friday Saidi, Twambilile Phanga, Jennifer Tseka, Agatha Bula, Pearson Mmodzi, Lisa D. Pearce, Suzanne Maman, Carol E. Golin, Wilbroad Mutale, Benjamin H. Chi, Lauren M. Hill

**Affiliations:** ^1^ Department of Maternal and Child Health, University of North Carolina at Chapel Hill, Chapel Hill, NC, United States; ^2^ Women’s Global Health Imperative at RTI International, Berkeley, CA, United States; ^3^ UNC Project-Malawi, Lilongwe, Malawi; ^4^ Department of Obstetrics and Gynecology, School of Medicine, University of North Carolina at Chapel Hill, Chapel Hill, NC, United States; ^5^ Department of Sociology, University of North Carolina at Chapel Hill, Chapel Hill, NC, United States; ^6^ Department of Health Behavior, University of North Carolina at Chapel Hill, Chapel Hill, NC, United States; ^7^ Department of Medicine, University of North Carolina at Chapel Hill, Chapel Hill, NC, United States; ^8^ Department of Health Policy, University of Zambia School of Public Health, Lusaka, Zambia

**Keywords:** HIV, PrEP, pregnant and breastfeeding women, Malawi, male partners, social support

## Abstract

**Introduction:**

Daily oral pre-exposure prophylaxis (PrEP) is a safe and effective HIV prevention method for pregnant and postpartum women, but adherence barriers exist. Understanding the role of male partners in supporting PrEP use may inform strategies to support PrEP adherence among pregnant and breastfeeding women.

**Methods:**

To understand male partners’ involvement in women's use of PrEP, we conducted in-depth interviews with pregnant women in Lilongwe, Malawi who had recently decided to use PrEP (*n* = 30) and their male partners (*n* = 20) in the context of a PrEP adherence trial. Women were purposively recruited to ensure variation in their partners’ HIV status. Interviews were conducted in Chichewa using a semistructured guide. We followed a thematic approach to analyze the interview data.

**Results:**

Most male partners were receptive to women using PrEP during pregnancy because it eased their fears of the woman and baby acquiring HIV. Men often played a key role in women's PrEP adherence by providing daily reminders and encouragement to adhere to their medication. The majority of women appreciated this support from the men as it lessened the burden of remembering to take their medications daily on their own and aided their adherence. However, several women who lacked male partner support spoke of wanting their partners to be more involved. Many men living with HIV found the mutual support beneficial for their antiretroviral therapy adherence, while men without HIV or with status unknown appreciated knowing that the family was protected. While most men were open to women continuing PrEP beyond the current study, some would only support it if women were still at risk for acquiring HIV.

**Conclusion:**

In this study, male partners were strongly motivated to support the PrEP adherence of their female partners as a way of ensuring that the pregnant women and unborn babies were protected against HIV. Promoting disclosure and tangible support that arises organically among men may be helpful, but programs to enhance this support and identify ways to support women who do not receive support from their partners or do not wish to disclose their PrEP use to partners may be needed.

## Introduction

Women in Eastern and Southern Africa face substantially elevated HIV risk during pregnancy and the postpartum period due to increased biological and behavioral risk factors (1–5). Acute maternal HIV infections are responsible for an estimated one-third to one-half of mother-to-child transmission (MTCT) (6, 7). Daily oral pre-exposure prophylaxis (PrEP) is a safe and effective method for preventing HIV acquisition when taken with high adherence (6–8).

To deliver on the promise of PrEP for MTCT, the World Health Organization guidelines recommend that oral PrEP be offered in standard Prevention of mother to child transmission practice (9). However, while many pregnant HIV-negative women are willing to initiate oral PrEP, early discontinuation and low adherence are common, especially in young pregnant women, and little is known about the potential facilitators of oral PrEP persistence in this population (10–13). Based on the broader medical literature, one potential factor—may be male partner support. Studies have suggested that direct engagement of male partners might play a role in women's adherence to HIV prevention technologies (14, 15). Women's disclosure of their HIV prevention products to their male partners and positive reactions from male partners have been reported to increase women's product adherence and facilitate partner adherence support (16, 17). In one clinical trial, women who had disclosed to social contacts (including male partners) had almost five times the odds of continuing oral PrEP at trial exit than women who had not done so (18).

Although studies have examined the use of oral PrEP during pregnancy from women's perspective, there is a gap in understanding the exact role male partners play in such decision-making from a dyadic perspective. A few studies have reported male partner support to be beneficial to women's PrEP use during pregnancy (10, 19); however, they did not explicitly describe how their support facilitated adherence or try to capture men's perspectives on how they provided support. Qualitative studies and the flexibility they provide are well suited to uncovering the types of support male partners provide and which types of support women find most helpful. Understanding the impact this support from male partners has on their adherence can inform strategies to help pregnant and breastfeeding women use PrEP at effective levels to help facilitate the development of better partner support programs. In this study, we used in-depth interviews to understand, from the perspective of both women and men, how male partners were involved in supporting women's oral PrEP use during pregnancy and postpartum and the impact this support had on their PrEP adherence. We further looked to understand the bidirectional impact of women's PrEP use on antiretroviral therapy (ART) use among male partners living with HIV.

## Methods

### Study context

The data presented here were collected as part of the Tonse Pamodzi 2 (TP2) pilot trial (20). The PrEP component of this study enrolled pregnant women, age 18 years or older, who were at risk of HIV acquisition and interested in initiating daily oral PrEP in Lilongwe, Malawi. Pregnant women living without HIV were eligible to participate if they met any of the following HIV risk indications for PrEP: having a known positive partner or an unknown partner HIV status, having multiple partners, having Sexually transmitted infections (STIs) diagnosis, using postexposure prophylaxis, or having an unspecified HIV risk concern; for full eligibility criteria, see (20). All women were counseled about their HIV risk and how PrEP could reduce it. Participants received basic HIV prevention education regarding the functions of daily oral PrEP, the importance of adherence, side effects, and safety. Women were prescribed PrEP at the enrollment visit and were given further details on PrEP dosage and efficacy, duration of use, and adherence strategies. Women were randomized 1:1 to either the standard support for PrEP or a combination adherence strategy that included Integrated Next Step Counseling and optional adherence supporter training. The intervention development process has been described in detail elsewhere (21).

### Recruitment and data collection

We recruited a subsample of women (*n* = 30) to participate in individual in-depth interviews (IDIs) to explore their perspectives of men's involvement in their PrEP use experience. In addition, male partners of the women (*n* = 20) were recruited to investigate their involvement in women's PrEP use. We purposively recruited women from the TP2 trial for this substudy to ensure variation in their partners’ HIV status. Male partner HIV status was reported by women during the baseline survey and was confirmed by the men during the IDI. Male partners (*n* = 14) were recruited through invitation by women participating in the substudy. When that recruitment approach was exhausted, additional male partners (*n* = 6) were recruited via invitation by other women participating in the parent TP2 PrEP trial. All male partners were aware of the women's use of PrEP.

Women completed the IDI an average of 102 days after enrollment (range: 59–239 days), and interviews lasted approximately 25–40 min. All IDIs were conducted in a private room at the study site in Chichewa by a qualitative research officer fluent in Chichewa and English using a semistructured interview guide. A male qualitative research officer conducted IDIs with male partners who were uncomfortable being interviewed by a female officer. Both men and women interviewed were asked about partner involvement in women's PrEP use and adherence support that men provided their partners. In IDIs with the women, we also sought to understand the impact of partner support on their adherence. Male partners were also questioned about their feeling and attitudes toward the women's present and future PrEP use and, for partners living with HIV, the impact of the women's PrEP use on their ART use. All IDIs were audio-recorded, transcribed, and translated into English.

### Analysis

A thematic approach was used to analyze the IDIs. The approach consisted of (1) reading transcripts in full and noting emerging themes; (2) creating a codebook including structural codes (corresponding to interview topics) and interpretive codes (corresponding to emerging ideas); (3) coding with 20% of transcripts double-coded by independent coders who reconciled discrepancies prior to further coding; (4) summarizing participant responses pertaining to each topic/code in matrices to facilitate summaries by topic and comparisons across participants (22); and (5) making dyadic comparisons (15 dyads and *n* = 30 total participants) of women's and male partner's narratives through combined matrices summarizing the different themes that emerged from the women and men, noting the differences in the narratives given by the women and male partners, and observing the variation within each theme by the HIV status of the male partner. Separate codebooks were used for men's and women's interviews with similar codes for similar interview questions and separate codes for interview questions unique to each participant group (e.g., questions for partners regarding the provision of adherence support). Coding was completed using the NVivo version 12 software tool (23).

### Ethical considerations

Study procedures were approved by the Malawi National Health Science Research Committee and the University of North Carolina at Chapel Hill Institutional Review Board. The TP2 pilot trial was registered on www.clinicaltrials.gov (NCT04330989). All participants provided written informed consent prior to study procedures. A literate impartial witness was present during the consent process for illiterate participants.

## Results

### Sample description

A total of 50 participants (30 women and 20 men) completed IDIs (Table 1). The median age of women interviewed was 25 years. Of the women interviewed, six had a partner living with HIV, 14 had a partner without HIV, and 10 were unaware of their partner's status. The majority of the women (*n* = 27) were identified as PrEP candidates because of an STI diagnosis, while a good portion also had a partner with an unknown HIV status (*n* = 8) or an HIV-positive partner (*n* = 7). A few women (*n* = 4) reported having more than one sexual partner. Moreover, at the time of the interview, 13 women were pregnant and 17 were in the postpartum period. Among the male partners who agreed to have an IDI, six were living with HIV, 10 were without HIV, and four did not know their HIV status. Finally, of the 50 individuals interviewed, 30 of them were part of dyads (*n* = 15 dyadic couples).

**Table 1:** Demographic description of participants (*N* = 50).

	*N* (%) or median (range)
Pregnant women (*n* = 30)	
Age	25 (18–40)
Reported partner's HIV status	
HIV-positive	6 (20)
HIV-negative	14 (46.7)
Unknown	10 (33.3)
PrEP eligibility reasons (past 12 months, not mutually exclusive)	
STI diagnosis	27 (90)
Partner of unknown HIV status	8 (26.7)
HIV-positive partner	7 (23.3)
Multiple sexual partners	4 (13.3)
Pregnancy status at the time of the interview	
Pregnant	13 (43.3)
Postpartum	17 (56.7)
Male partners (*n* = 20)	
HIV status	
HIV-positive	6 (30)
HIV-negative	10 (50)
Unknown	4 (20)
Dyadic couples	30 (60)

### Male partner feelings toward women’s PrEP use

Overall, male partners reported being happy that women were using PrEP because it reduced their fears of women contracting HIV. They welcomed women's PrEP use, although some misconceptions about the benefits of PrEP were observed. The men rarely reported negative feelings regarding the time the women were using PrEP; however, some did express initial concerns that PrEP use could affect the unborn fetus. Only one partner mentioned concerns regarding his partner experiencing weakness in the morning; however, he was unsure whether it was caused by PrEP or part of the pregnancy symptoms. These initial negative feelings or concerns were usually resolved as the women continued with PrEP use or through study staff support.

#### Reduced fears

Most men expressed happiness that women were using PrEP as their use alleviated some fears of the woman and child acquiring HIV. Many men feared that their partners were at risk for contracting HIV because of the STI diagnosis; thus, some saw women's use of PrEP as a way of protecting the health of the entire family.

*“I liked it [PrEP] because it helps my wife’s immunity, she is the one that is going to be bearing children for me so this will affect my unborn babies…When I understood that the PrEP is helping so that the mother should not be infected that meant that my baby would also not get infected that is why I said continue taking the medication.” [Male partner, HIV-negative]*

This was especially salient among men living with HIV that were now comfortable having sex with their partners as they were no longer concerned about potential HIV transmission, as illustrated by one man:

“*What I am loving is that when it’s time for us to be intimate there are no problems because she would have already taken the medication so there are no fears,” [Male partner, HIV-positive]*.

#### Misconceptions of PrEP benefits

Among the men, there were misconceptions regarding the actual function of PrEP; however, in most cases, these misconceptions positively influenced men's feelings about women's PrEP use. Some men thought women's PrEP use had improved women's health by strengthening their general immunity as the women were no longer falling sick frequently or perceived the women's physical appearance to have improved while on PrEP. Two male partners illustrate this point below by contributing their partner's improved overall health to their PrEP use:

*“…at the time that she had not started the PrEP she was one that was often sick but now I see that everything has changed meaning that its good…I never expected that there would be medication like this that would make the body better. She used to be complaining every day. [Male Partner, HIV-positive]*

*“The way she takes the medication and her body looks good…She looks good and it shows that she is strong…from the time that she started taking the medication…Ok let me put it like this, before she used to have a malnourished body…Now her body is healthy…Meaning that what [PrEP] she is taking is helping add energy to her body.” [Male Partner, HIV-unknown*]

A few of the men also thought PrEP simultaneously treated the STIs that some women had been found to have at the initiation of the study or that it would prevent future STIs. This misconception that PrEP was preventing more than just HIV or improved overall health was also shared among the women, who, like the men, thought they were now protected against other STIs. In the quote below, a woman explains her belief that PrEP use would treat her STI diagnosis and further protect her from future recurrences of STIs:

*“From that time…the PrEP was supposed to help me from STIs that I was found with, so it helped to reduce the infection and to protect me.” [Woman, Partner HIV-negative]*

While most men welcomed women's use of PrEP, it was challenging for one man to accept it as he did not understand the point of taking preventive medicine when not sick. To him, medication was reserved for when an individual was sick and wanted to improve their health condition. He did not understand the concept of “treating” something you did not have, as the effect of this medicine is not visible since the individual is already healthy:

*“What I can say is that it is different from a person that is sick. It is like when a person is sick there are certain goals that you want to achieve which are for the person to recover. But for PrEP it’s like the person just takes daily and you don’t really see the goal that you want to achieve.” [Male partner, HIV-negative]*

### Male partner’s involvement in women PrEP adherence

Male partners provided adherence support to the women in the form of reminders, motivation, strategy development, and instrumental support. This support was confirmed by most women who agreed that their male partners played a role in providing adherence support; however, some women spoke of their male partners not being involved in reminding them to take their medication. The ways in which partners supported women's PrEP use are discussed below.

#### Adherence support

Most men spoke of playing a key role in supporting women's PrEP use by providing daily reminders. On rare occasions, as characterized by the male partners, when women were struggling with adhering to their medication, some men went beyond just giving reminders and became motivators by encouraging the women to stick to the daily regimen, as illustrated by the quote below:

*“So, you know maybe she is not in a good mood but you are still supposed to force her to take the medication, those are the major challenges but then I am thankful that the medication has been taken and she has completed them.” [Male partner, HIV-negative]*.

Some men also helped women come up with adherence strategies which included the men setting alarms to remind the women:

*“I put an alarm on my phone so when that goes off I know that it’s now time… It also happens that maybe I am still in town I just call her to remind her because it’s not always that I get here at a good time sometimes I knock off late.” [Male partner, HIV-negative*]

Other men provided instrumental support to their wives to take PrEP daily, such as bringing the pills and water to the women at their dosing time:

*“My wife here is a cup of water and medicine for you to take. Don’t bother moving out of where you are sitting. Just take the medicine.” [Male Partner, HIV status unknown]*.

In a unique case, one man spoke about reminding his wife to take her medication and even coming up with different signals (phrases) for the woman to give each other when in public or around other people to indicate when it was time for the woman to take her PrEP. He explained in the quote below how he reminds her and the different phrases he uses to signal it is time for her medication:

*“I tell her to come and then remind her that it’s time to take her medication, if she is in a group, I remind her. When we are in public there are signals that we give each other….I even tell her to go get me a cup of water or prepare my bath water and she knows that it’s time to take the medication.” [Male partner, HIV-positive]*

Men not only played the role of supporter or motivator when they were physically with the women (e.g., at home) but even when they were away from home either because of traveling or working late. A few still called the women to ensure they had taken their PrEP.

*“I considered the time that she set to be taking the medicine and the time I knock off from work, sometimes I arrive home late so it because hard to be waking her up to take the medicine so I just call her to take the medicine.” [Male partner, HIV-negative]*

While others, especially those who traveled often for work, reinforced the importance of adhering to the medication before leaving home. In the quote below, one woman talks about how her husband often travels part of the month for work and that while she was using PrEP, he always encouraged her to adhere to her medication while he was gone:

*“He tells me to not forget to take the medication because his business involves him being away for around 2 weeks at the lake before coming home. He encourages me when he is going that I should not forget to take the medication when he is gone.” [Woman, Partner HIV-positive]*

Reminders were not only for PrEP adherence but also included clinic reminders when it was time for the women's next visit. In a way, this ensured women received the essential PrEP refills needed to continue their adherence and protection. As previously stated, some men viewed women's decision to use PrEP as a family affair and not solely the PrEP user themselves, which meant everyone was involved in ensuring high adherence:

*“We did this because we agreed in the home as a family that is why I chose to play a part by reminding her so that when her scheduled day [clinic appointment visit] is there she should be coming [to the clinic].” [Male partner, HIV-negative]*

The information above illustrates the key role that men played in assisting women with PrEP adherence. This narrative was reinforced by the women who agreed that the support from the men positively assisted with their PrEP use.

*“…My husband tells me to be taking the medication… He says that I should be taking the medication so that it should be protecting me from the disease.” [Woman, Partner HIV-positive]*.

*“… it is the person who reminds me to take my medicine, that’s my husband, he is the one who encourages me to take my medicine with good adherence and on time.” [Woman, Partner HIV-negative]*

#### Lack of support

Although most women received adherence support from the men, it was not the case for all. A few women spoke of not receiving any support from the men; however, these women motivated themselves to adhere to the medication regardless of the lack of support from the men because they wanted to protect themselves and their unborn children.

*“There is nothing that they [my partner] do I just remember by myself.” [woman, Partner HIV-positive]*

Indeed, in one dyadic relationship, one man spoke of being involved in the initial decision-making for the woman to use PrEP; however, he was not participating in her adherence because he felt she was handling the situation well and, therefore, did not feel compelled to provide encouragement.

*“She adheres and even if I get home and she has finished taking the medication you see her checking her phone to check the time, she was apparently told the time that she should be taking the medication…I don’t help her she just knows that it is now time for me to take PrEP.” [Male partner, HIV-negative]*

Although the man in this case felt he was supportive of his partner's use of PrEP, the woman felt she was not supported because the support was not explicit, and she would have preferred encouragement and reminders.

“*[I would like him] to be checking if I have taken the medication and if not be encouraging me to be taking.” [Woman, Partner HIV-negative]*.

In another dyadic relationship, one man living with HIV spoke of encouraging his partner by setting an alarm and often giving her transportation means for clinic visits; however, his account appeared to be contradicted by his partner's account, who said she was her own support. Other women who spoke of not receiving support from male partners were in non-dyadic relationships, and thus their partners' perspectives were not included as they did not participate in the study. Men did not express concerns about women struggling with PrEP adherence but rather felt it was important to provide moral support to show the women they supported their PrEP use. All women in the study, except for two women, disclosed their PrEP use to their male partners. For the two women who did not disclose, reasons for non-disclosure included the following: (1) male partner passing away right before she joined the study; and (2) no longer being in a relationship with the male partner.

### Impact of male partner support on women’s PrEP adherence

While the majority of women interviewed could not think of the ways in which men's support (e.g., reminders and encouragement) directly contributed to their adherence, a few of them spoke of the impact of the men's support on their PrEP use. These women felt the support provided by the men influenced their overall adherence as it ensured they took the medication on time and provided them a sense of comfort knowing that they were not on the PrEP journey alone.

*“At times when I forget, such as if I just wake up and start working, you know one is just human and can forget, he reminds me to take my medication before I start working… I feel so good!…Yes, I feel that we are together in this journey.” [Woman, Partner HIV negative]*.

Some men provided additional support by addressing women's concerns about using PrEP, especially as it pertains to potential future adverse effects on the unborn child. In one case, one man spoke of encouraging the woman not to listen to rumors from friends that PrEP caused her miscarriage and was “satanic.” He reached out to the study staff, who counseled the woman once he realized she was still discouraged and planned on dropping out of the study. The woman affirmed that her partner's involvement ensured that she continued with study participation and product use because had he not called the doctor, she would have discontinued PrEP. In the quote below, she explains the role her partner played in ensuring she continued with study participation:

*“… So I told him [doctor] everything that happened and he encouraged me there. He [doctor] came because my husband called him on the phone telling him ‘My wife has called me saying she is dropping out of the study for such and such reasons. So, I want to come there so you can explain to her because I have encouraged her but she doesn’t look convinced.’ That’s when they called me to come here and the doctor talked to me and encouraged me so I understood.” [Woman, Partner HIV-negative]*

Women were generally satisfied with the support they received from their partners, although some wanted men to be more involved in their PrEP journey, including escorting them to clinic visits. There were no discrepancies in the direction of women citing support and the men saying they did not really provide any support.

### Impact of women’s PrEP use on men’s ART

The male partners who were living with HIV (*n* = 6) spoke of the domino effect of the women's PrEP use on their ART adherence as they were able to remind each other when it was time to take their medication. Most of these men gave the impression that women's use of PrEP improved their ART adherence through encouragement and mutual support*.* The quote below showcases a collaborative effort between the male partner and the woman to ensure they both take their respective medications at the appropriate times, highlighting the communication and support within their relationship regarding HIV prevention and treatment.

*“I just tell her to take [her PrEP], or I just take other times [sometimes he just gives the woman her PrEP when he gets his own ART] I just tell her that it’s time for us to taking the medication,” [Male partner, HIV-positive]*.

The woman affirmed this man's narrative and added:

* “On the issue of medication, we did not discuss anything because it is him who encourages me when it is time to take my medication and he takes his too,” [Woman, Partner HIV-positive]*.

The concept of the women's PrEP use being a family affair also emerged when some men discussed how it impacted their ART adherence. One man spoke of his children getting involved with their PrEP and ART adherence.

*“She also reminds me that you should be taking the medication as we have both been told to be adhering to the medication. We have now reached the point of getting used to the extent that we even send the children to get the medication for us,” [Male Partner, HIV-positive]*.

Men spoke of adhering to their medication because they understood it would improve their quality of life, prevent them from future health issues, and, most importantly, decrease the likelihood of women and future children acquiring HIV. There was no indication of decreased motivation among the men on the basis of women's PrEP use as they understood that adhering to their ART the same way the women were adhering to their PrEP would ensure continued protection of the woman and baby and further recognized that PrEP and ART were two distinct medications.

*“I am encouraged and she also frequently reminds me…You just feel that if you skip the medication, you can develop a problem in your body…That is why I try to be taking the medication daily.” [Male Partner, HIV-positive]*

*“Yes, I adhere [to my ART]… Because the medicine is different the ARTs and PrEP are different so I should not take advantage of that so I stop taking the medication, no… Its better I be taking the ARTs and she also be taking her medication.” [Male Partner, HIV-positive]*

### Men’s thoughts on women’s future PrEP use

The desire for women to stay protected even after the conclusion of their participation in the TP2 trial by continuing their PrEP use was supported by a majority of men, especially since there were no observed negative side effects on the women during use. Some men felt that women's discontinuation of PrEP use upon study conclusion would lead to a worsening of health issues (e.g., immunity, STIs). Men living with HIV, in particular, worried that women's discontinuation of PrEP could potentially lead to the men transmitting HIV to the women and thus believed, as illustrated in the quote below, that it was vital for women to continue using PrEP, while the men continued with ART. Furthermore, these men worried that with PrEP discontinuation, women would revert to their previous health status (e.g., weak immunity, stomach pains) before they initiated PrEP:

*“Because it’s possible that she could stop taking PrEP while am continuing to take the ARTs. I feel that it’s important that she continues because it’s possible she could stop which could lead to problems in the future like she used to complain of…The issues that she used to complain of like sometimes she would feel pain in the stomach, other times maybe just eat a little I feel that if she stops these issues could reoccur.” [Male Partner, HIV-positive]*

Similarly, men without HIV or whose HIV status was unknown felt that women’s continuation of PrEP beyond the study would safeguard the family as HIV could be acquired in various ways.

“*I would encourage her because we can face different situations in life, so in order for us to protect each other she can still be taking the medication… I can say that people contract HIV in many ways so I would encourage her to be taking PrEP as a way of protecting her.” [Male Partner, HIV-negative]*

Only a few men felt they would only agree to women's use if they or the woman felt it was appropriate and that the women were at risk for acquiring HIV (e.g., future STI diagnosis). This is not to say these men were not currently supporting the women with PrEP use; however, it came across as if they felt that since the women had already been treated for STIs, they would not necessarily need to continue PrEP due to low susceptibility to HIV. One male partner illustrates this opinion in the quote below, saying that he would not allow his partner to continue using PrEP because she would have finished her STI treatment and was no longer at high susceptibility for HIV:

*“For her she cannot continue since I thought after she has taken all her medicine as per prescription then she doesn’t have to keep on taking them.” [Male Partner, HIV-negative]*.

This further illustrates a misunderstanding that some men had in that they believed the treatment of STIs implied women were no longer at risk for HIV. Moreover, only one man mentioned the cost of PrEP potentially being a barrier to the woman's PrEP continuation and advocated for it to be given out freely.

## Discussion

The study findings suggest that male partners can play an important role in supporting women's PrEP use and adherence to reduce the risk of HIV transmission to both mother and unborn child. Several women reported unsupportive male partners regarding PrEP use, despite desiring their involvement, emphasizing the need to engage disinterested partners. Involving male partners in identifying and implementing PrEP adherence strategies, such as providing motivation and reminders, may prove helpful in supporting pregnant women's adherence to PrEP as suggested by our findings. For male partners living with HIV, the study suggests that promoting women's oral PrEP use may positively affect their ART use through shared motivation and mutual support. Finally, while most male partners were supportive of women's PrEP continuation outside the study setting, a few were reluctant due to a perceived lack of HIV risk.

Most women interviewed received support from their male partners in the form of reminders and encouragement to take PrEP; however, only some were able to explicitly comment on how partner support was vital for their adherence to the medication. Receiving practical and emotional support, especially from male partners, in the form of reminders, encouragement, reassurance, and management of side effects has been reported in other studies as an important driver of consistent PrEP use (12, 24, 25). This suggests that interventions designed to increase women's PrEP uptake and adherence should consider the role of male partners and encourage their involvement in HIV prevention efforts, particularly when it comes to women's PrEP adherence. In addition to those reported in our study, other types of support from partners that women in other settings have deemed helpful include demonstrating interest in women's clinic visits (often asking what had transpired during visits), observing drug doses, doing pill counts, assisting with housework, and providing financial support for transportation to clinics for visits or refills (11, 25, 26). Women in our study illustrated the myriad ways support from male partners was instrumental in assisting women with their PrEP adherence, though primarily in the form of instrumental support. Moreover, some women who reported a lack of support from their male partners expressed a desire for their partners to actively participate in their daily adherence to PrEP and accompany them to clinic visits. Conversely, male partners expressed a willingness to provide increased support in the future by being more involved in the women's decision-making phase regarding the use of PrEP. A recent study in South Africa emphasized women's desire for increased male partner support through participation in HIV testing and counseling, aiming to enhance engagement in antenatal care services (27). Future interventions should seek to leverage the natural ways in which partners provide support while promoting other types of social support from partners or other sources, such as emotional and informational support (21), which may confer additional benefits for PrEP adherence (15, 28). Furthermore, healthcare providers should encourage women to involve their partners in their PrEP use and provide education to both partners about PrEP and its importance for preventing HIV transmission.

Most women in our study had disclosed their PrEP use to their male partners, and the men interviewed displayed good knowledge of PrEP's function. Yet, some men had misconceptions about oral PrEP's benefits, and—despite the incorrect knowledge—this sometimes motivated their support for women's use of PrEP. Although men had some initial concerns that PrEP could negatively affect the unborn fetus, more information from the woman or study staff usually alleviated these concerns. Partner PrEP education and involvement in initial counseling before women's PrEP use is essential to address partner PrEP knowledge about PrEP to facilitate appropriate partner support. By involving male partners in PrEP education and counseling, healthcare providers can ensure that both partners have accurate information about PrEP and address any concerns they may have (15, 29, 30). Future research should explore the effectiveness of different approaches to involve both partners in PrEP education and counseling, such as group sessions, couples, or individual counseling. Additionally, it is necessary to research on why some men, who do not have HIV but support women's PrEP use, may be motivated to protect the baby rather than the woman herself. Furthermore, a better understanding of how this primary motivation may impact these men's attitudes toward women's use of PrEP postpartum and after weaning is crucial.

The male partners living with HIV who we interviewed appreciated knowing the women had added protection outside their ART use and reported a perceived benefit in the mutual support for ART use. Shared reproductive health goals among couples, such as protecting the unborn child from HIV acquisition through oral PrEP, can promote mutual adherence (19, 31, 32). Evidence from other studies suggests that having oral PrEP as an option for women may help male partners living with HIV by giving them time to accept their HIV status, initiate ART, and create a feeling of being on the journey together by taking their medication at the same time (31, 33). For the PrEP-using partner, evidence suggests that matching dosing schedules with the partner taking ART may help to support their adherence to PrEP (25). Partner support programs are needed to facilitate this mutual ART-taking and support among serodiscordant couples to realize these many apparent benefits. Such programs might entail coeducation and training on supporting each other's ART use and joint adherence counseling. These programs may be particularly suitable in cases where the partner living with HIV is newly diagnosed, has not achieved viral suppression, or if the woman is uncertain about their partner's viral suppression status.

Our results illuminate the role that male partners can play as key supporters of oral PrEP use to promote prevention-effective oral PrEP use among pregnant and breastfeeding women. These results should be interpreted with key limitations in mind. First, participants' reports of male partner involvement and support of women's PrEP use and representations of PrEP adherence may be susceptible to social desirability bias. Participants were reminded that there were no right or wrong answers and that their responses would not affect their participation in the TP2 PrEP study or their relationship with the study clinic to minimize this bias. Second, male partners were purposively recruited primarily through women's referral; thus, more supportive partners may have been recruited; their experiences may differ from those of male partners who were not invited to be interviewed or were invited but did not enroll. Third, participants were recruited from urban and periurban areas; thus, the results could not be generalized to the larger Malawian population because perspectives and experiences might differ among women and men in rural areas. Finally, nearly all women who agreed to be interviewed had disclosed their PrEP use to their partners and thus cannot offer insight into the experiences of women who did not disclose their partners or declined to be interviewed. Future studies are needed to better understand the experiences of women who cannot or do not wish to disclose their PrEP use to their partners to identify alternative support strategies for women lacking partner support or to support partner disclosure of PrEP use if deemed appropriate.

## Conclusion

In our study, male partners generally supported women's use of oral PrEP during pregnancy, often motivated by the perceived desire to protect their unborn child from acquiring HIV. Men provided support in the form of reminders and encouragement for women's PrEP use and adherence. For male partners living with HIV, women's use of PrEP did not negatively impact their ART use; rather, the mutual support was viewed as enhancing by both parties. These findings emphasize the need for interventions to increase women's PrEP uptake and adherence to consider the role of male partners and encourage their involvement. This could lead to more successful HIV prevention outcomes for women and promote mutual support between partners to collaboratively address HIV prevention. Finally, policymakers and health practitioners could consider providing information on the importance of PrEP use for HIV prevention, regardless of perceived HIV risk, and involving male partners in promoting its use to reduce transmission rates.

## Data Availability

The raw data supporting the conclusions of this article will be made available by the authors without undue reservation.
